# Validity and reliability of the Setswana translation of the Short Form-8 health-related quality of life health survey in adults

**DOI:** 10.4102/hsag.v23i0.1092

**Published:** 2018-11-22

**Authors:** Sunday O. Onagbiye, Sarah J. Moss, Melainie Cameron

**Affiliations:** 1 Physical Activity, Sport and Recreation Research Focus Area, Faculty of Health Sciences, North-West University, South Africa; 2 School of Health and Sport Sciences, Faculty of Science, Health, Education and Engineering, University of the Sunshine Coast, Australia; 3 Redcliffe Hospital, Queensland, Australia

## Abstract

**Background:**

The absence of culturally relevant measures in indigenous languages could pose a challenge to epidemiological studies on health-related quality of life (HRQoL) in developing nations.

**Aim:**

To explore the feasibility and determine the validity and reliability of the Setswana translation of the HRQoL Short Form-8 (SF-8) among Setswana-speaking adults.

**Setting:**

Potchefstroom in the North West province.

**Methods:**

Sixty healthy men (*n* = 26) and women (*n* = 34), aged 45.5 ± 9.3 years, completed a Setswana translation of the SF-8 questionnaire and the original English version twice, with a 4-week interval between completions.

**Results:**

The Setswana SF-8 presented good concurrent validity with the Spearman’s correlation coefficients (*ρ*) of 0.72 for role physical to 0.91 for social functioning. The Cronbach’s alpha coefficients for the first and second measurements were 0.87 and 0.87, respectively, for the Setswana-translated SF-8 and 0.86 and 0.89 for the original English SF-8. The reliability coefficients were moderate for the mental health (*ρ* = 0.60), social functioning (*ρ* = 0.56) and role emotional (*ρ* = 0.50) domains, as well as the mental component summary (*ρ* = 0.50) and physical component summary (*ρ* = 0.45), but fair for the role physical (*ρ* = 0.43), body pain (*ρ* = 0.43), general health (*ρ* = 0.42), physical functioning (*ρ* = 0.41) and vitality (*ρ* = 0.38) domains on the translated Setswana version of the SF-8.

**Conclusion:**

The Setswana SF-8 version was feasible, acceptable and had acceptable concurrent validity and fair to moderate evidence of test–retest reliability for assessing HRQoL among adult Setswana-speaking community dwellers.

## Introduction

Over the past 20 years, the interest in health-related quality of life (HRQoL) has increased because of the concept’s potential to facilitate meaningful improvements in health services and policy research (Ruževičius & Akranavičiūtė 2007). The increase in interest has contributed to HRQoL being internationally accepted as a valid indicator of health status. This global acceptance means that it has the potential to be a valuable mechanism to compare and aggregate results of efficacy and effectiveness studies across countries and groups. To facilitate such international comparison and aggregation, the concept needs to be meaningfully operationalised across different language and cultural groups. The current study is an example of one such meaningful cross-cultural operationalisation (Ruževičius & Akranavičiūtė 2007).

HRQoL evaluations are used as health indicators to monitor the health status of populations and for programme appraisal (Al Sayah et al. 2013). The personal attributes of understanding the meaning of life could help people look for a higher existential level of their life (Ruževičius & Akranavičiūtė 2007). This can be achieved by understanding the individual’s satisfaction with life dimensions compared to an ideal life, which depends on one’s value system (Ruževičius & Akranavičiūtė 2007). HRQoL could also be dependent on external factors that could influence the living conditions of individuals (Ruževičius & Akranavičiūtė 2007). These external factors, which could influence the HRQoL, could include accommodation, employment status, income, welfare, moral attitudes, family life, social support, stress and crisis, education and environment, among others (Ruževičius & Akranavičiūtė 2007).

The concept of HRQoL is in general use and has cut across different healthcare fields, with the major aim to clinically improve the current health status of people and communities at large (Boixados et al. 2009). It is an all-inclusive concept with a complete understanding of health and its various meanings (Ellert & Kurth 2013). The approach of HRQoL based on this study was from the paradigm of healthcare and well-being. Meanwhile, it has been approved by professionals in the field, in that HRQoL possesses important components, which include the physical, emotional, mental, social and behavioural-related components of well-being (Ellert & Kurth 2013). The Short Form-8 (SF-8) has become an accepted and decisive instrument to clinically determine health status among individuals (Ellert & Kurth 2013).

The SF-8 is seen as a standard instrument that is easy to administer within a very short period of time, as reported by this classic source (Ware et al. 2001). It is a general survey instrument that can be used to clinically assess health status regardless of age, illness and treatment (Roberts et al. 2008). The SF-8 health survey can also be administered by mode of interview, most especially among people with low educational qualifications (Ware et al. 2001). Turner-Bowker et al. (2013) found that the SF-8 could be used to access HRQoL in a group of people suffering from migraines and other chronic ailments. Compared with the Short Form-36 (SF-36), which was extensively used, the SF-8 survey achieved adjusted multiple correlations of 0.88 and 0.83 in the prediction of the mental component summary-36 (MCS-36) and physical component summary-36 (PCS-36), respectively (Lefante et al. 2005). The reason for using the SF-8 was established after the slow response from the participants because of time constraints in responding to the 36 questions in the SF-36 health survey instrument (Lefante et al. 2005). Considering the different sociocultural and physical environments from populations in other parts of the world compared to persons from sub-Saharan Africa, mere translation of a questionnaire may not be adequate to maintain content validity (Oyeyemi et al. 2011). Meanwhile, it is important to produce translated versions that do not lack experimental and theoretical similarity (Oyeyemi et al. 2011).

In cultural terms, adapting a questionnaire, rather than developing a new questionnaire, could be economical and could facilitate future comparisons among populations. The SF-8 has been translated into many languages with good reliability including English adaptation (Lefante et al. 2005; Turner-Bowker et al. 2013). Culturally, it is important that this health survey be translated into more languages in order to reach full understanding (most especially where English is not a local or official language) and be able to compare the results across different language and cultural groups and from one country to another.

The Setswana language is one of the local and official languages, spoken by 3 million Setswana people, one of the larger black minorities who span South Africa, of which the majority reside in the North-West province and Botswana (South African History Online 2015). However, the translated Setswana version of the SF-8 and aspects of validity and reliability have not been demonstrated in this population. The purpose of this study was to translate and cross-culturally adapt the original South African SF-8 and to evaluate aspects of validity and reliability of the Setswana-translated and culturally adapted version of the SF-8.

## Research design and methodology

The aspect of validity and test–retest reliability of the Setswana-translated and English versions of the SF-8 was assessed by repeated administration of the questionnaire with a 4-week interval.

### Participants

A convenience sample of 60 participants was recruited among community dwellers in Ikageng, a low-resourced area in Potchefstroom, North West province of South Africa (SA). Healthy Setswana-speaking men and women, aged 35–65 years, were included in the study. Persons were considered apparently healthy when they were not taking any chronic medication or suffering from any known diseases or lung conditions. All the participants were requested to complete an informed consent form before participation in the study.

### Measuring instruments

#### Demographic information

A demographic tool was used to elicit information regarding the participants’ date of birth, age, sex, marital status, employment status, level of education, type of house in which they lived and household income and was completed by the participants themselves.

### Questionnaires

#### SF-8 health-related quality of life survey

The SF-8 questionnaire, referred to as the SF-8, was selected based on its simplicity for the administrator and participants, easy translations, cultural adaptation and the establishment of psychometric properties (Roberts et al. 2008). The SF-8 consists of eight questions that measure the following broad domains: general health (GH), physical functioning (PF), role physical (RP), bodily pain (BP), vitality (VT), social functioning (SF), role emotional (RE) and mental health (MH). It has two component measurements, namely physical component summary (PCS) and mental component summary (MCS). The scoring in SF-8 was based on this two-component summary and was calculated by weighting each SF-8 item using a norm-based procedure in the instrument guidelines. The SF-8 was a five- and six-item Likert scale questionnaire, of which one response per question could be selected (Ware et al. 2001). The original South African English SF-8 was obtained from Quality Metric Incorporated (an Optimum Insight Company; USA) with licence number QM021030.

### Translation and cultural adaptation

The SF-8 was translated and adapted according to forward and backward translation guidelines (Guermazi et al. 2012). Two separate translations into Setswana were made by two native Setswana persons with more than 5 years of experience in the process, both of whom were fluent in both English and Setswana and experienced in translation. The SF-8 was then translated back into English by two students, one Psychology student, and the other from the Setswana department of North-West University. Both translators were fluent in Setswana and English and also experienced in translation. The translators were familiar with the purpose of the translation as well as the concept and constructs of the SF-8. The translation and back-translations were compared and inconsistencies resolved by consensus. To determine the ideal and item sameness, a correction group was consulted for the appraisal of the back-translated questionnaire, which was conducted by one of the study team members. The purpose was to make sure that the meanings and the concepts of the questionnaire items remained the same (Roberts et al. 2008). The study team member who appraised the translation was also fluent in both the Setswana and English languages. The participants were requested to complete the questionnaire twice exactly 4 weeks apart. A day interval was allowed during the data collection between the translated Setswana version and the English version. The Cronbach’s alpha correlation coefficient was set at above 0.70 for reliability.

### Pretesting of the Setswana Short Form-8 translated questionnaire

Pretesting of the Setswana-translated SF-8 questionnaire was done to check accuracy of translation and was piloted on a sample of Setswana-speaking people. Twenty participants who were not part of the main survey were selected based on convenience sampling from the same area from which the participants were to be recruited. Furthermore, answers to the translated questionnaire were provided by these participants in order to verify if the questions were acceptable or not (Guermazi et al. 2012). A group review was held by the study team, who were also part of the data collectors used for pretesting to check for errors or inconsistencies (Roberts et al. 2008) and who were fluent in both Setswana and English. Finally, the current Setswana version of SF-8 was produced based on the forward and backward translation, with a final review conducted by the study team (Roberts et al. 2008). Based on the fact that the questions were responded to without any difficulty it was assumed that the participants understood the questions (Roberts et al. 2008).

### Psychometric properties of the scale

The psychometric testing of the Setswana-translated version of SF-8 was based on data collected from a sample of 60 participants.

### Face validity

The satisfactoriness of the items (as well as their acceptability) was studied, while the time needed to complete the questionnaire was noted (Guermazi et al. 2012).

### Procedure for the administration of questionnaires

Before data collection, permission to administer the questionnaires was granted by the Department of Health, North West province. Information flyers inviting community members to participate in this research study were distributed in the community via churches and recreation facilities where community members meet on a regular basis. Upon arrival at 09:00, the participants were allowed to gather inside the community hall. Letters of informed consent were distributed and the purpose of the study explained. Participants interested in partaking in the study returned the following day and signed the informed consent letter. The purpose of the Setswana-translated SF-8 HRQoL survey was explained to the participants in their mother tongue by the team of researchers, who were well grounded in communicating in the Setswana language. Demographic information was completed within 5–10 min by each participant. The SF-8 was then completed by each participant in private within 5 min. The participants were asked to place the completed surveys in a holder near the door upon leaving the room. Participants who needed clarification on some of the questions were assisted by the researcher supervising the process. The participants were requested to return the following day in order to complete the English version of the SF-8. After completion of the English version of the SF-8, participants were requested to return in 4 weeks, at which time the surveys were repeated by all participants again 1 day apart for the Setswana and English. A token of appreciation was given to each participant who returned to complete the questionnaire 4 weeks later.

### Data analyses

Data analyses were carried out using the Statistical Package for Social Sciences (SPSS) 22.0 (IBM Corporation NY) statistical software package. All statistical tests were two-tailed and *p* *<* 0.05 was considered statistically significant. Descriptive statistics, such as mean, standard deviation and frequencies reported as percentages, were used to examine the demographic information of the participants. Internal consistency was assessed using the Cronbach’s alpha coefficient. A coefficient alpha of 0.70 or greater was generally considered to be acceptable (Guermazi et al. 2012). Test–retest reliability was estimated by calculating the Spearman’s rank correlation coefficient. Furthermore, the concurrent validity was also assessed using the non-parametric Spearman’s rank correlation coefficient. Spearman’s rank coefficient values were interpreted as follows: excellent relationship *ρ* ≥ 0.91; good relationship *ρ* = 0.90–0.71; moderate relationship *ρ* = 0.70–0.51; fair relationship *ρ* = 0.50–0.31 and little or no relationship *ρ* ≤ 0.30 (Guermazi et al. 2012).

### Ethical considerations

Approval for the study was received from the Health Research Ethics Committee – Humans, Faculty of Health Sciences of North-West University, Potchefstroom Campus (NWU-00002-14-A1).

## Results

The basic demographic characteristics of the participants in this study are presented in Table 1. A sample of 60 (men, *n* = 26, and women, *n* = 34) participated in the study. The mean age of the participants in the study was 45.4 ± 9.4 years with an age range of 35–65 years. More than half of the participants were female (58.3%). The mean years of working were 14.2 ± 11.1 years. The majority of participants were not married (38.3%) and not working (56.7%). The majority of the respondents (60%) completed high school, while 56.7% were unskilled. The largest percentage of the participants (63%) earned less than 1000 rand every month, while 35% earned between 1000 and 5000 rand, and 1.7% more than 5000 rand per month, per household. More than half (58.3%) of the participants lived in a brick house, while 1.7% lived in a flat or apartment and the remaining 40% in an informal type of house (shack).

**TABLE 1:** Demographic characteristics of the participants.

Demographic variables	Mean (SD)	*Frequency* (%)
**Age**	45.4 (9.39)	
**Gender**		
Men		26 (43.3)
Women		34 (56.7)
**Race**		
Black		57 (95.0)
Mixed race		3 (5.0)
**Marital status**		
Never married		23 (38.3)
Currently married		16 (26.7)
Living with partner		14 (23.3)
Widowed		2 (3.3)
Separated		4 (6.7)
Divorced		1 (1.7)
**Education level**		
None		3 (5.0)
Primary school		20 (33.3)
High school		36 (60.0)
College/university		1 (1.7)
**Current employment**		
Full-time		14 (23.3)
Shift		4 (6.7)
Part time (>10 hours/week)		1 (1.7)
Casual work		7 (11.7)
Not working		34 (56.7)
**Occupation level**		
Professional		4 (6.7)
Skilled		22 (36.7)
Unskilled		34 (56.7)
**Net income per month**		
< R1000		38 (63.3)
R1000–R5000		21 (35.0)
> R5000		1 (1.7)
**Type of residence**		
Brick detached housing		35 (58.3)
Brick cluster living		1 (1.7)
Informal (shack)		24 (40.0)
**Years of work**	14.2 (11.1)	

SD, standard deviation.

### Translation

The forward translation of the SF-8 was carried out by two people, with its synthesis leading to a unique version. Furthermore, the two backward translations of this version of the SF-8 were compared to the original scale (Guermazi et al. 2012).

### Pretesting

Twenty participants in total, with the mean age of 47 years (min. 35 and max. 64 years) participated at this stage, after which minor corrections were made to the instrument and a final translated version of the SF-8 was acquired.

### Metric properties of the scale

Sixty participants (26 men and 34 women) answered the questionnaire twice (4-week recall). Table 2 shows the test–retest reliability of both translated Setswana and original English version of the SF-8 items.

**TABLE 2:** Test–retest reliability of Short Form-8 items in Setswana and South African English.

Variables	*ρ*	*p*	*ρ*	*p*
Q1 – General health	0.42*	0.001	0.51*	0.000
Q2 – Physical functioning	0.41*	0.001	0.42*	0.001
Q3 – Role physical	0.43*	0.001	0.50*	0.000
Q4 – Bodily pain	0.43*	0.001	0.44*	0.000
Q5 – Vitality	0.38*	0.003	0.52*	0.000
Q6 – Social functioning	0.56*	0.000	0.53*	0.000
Q7 – Role emotional	0.50*	0.000	0.51*	0.000
Q8 – Mental health	0.60*	0.000	0.61*	0.000
PCS	0.45*	0.000	0.63*	0.000
MCS	0.50*	0.000	0.61*	0.000

* , correlation is significant at *p* ≤ 0.01 levels (two-tailed).

PCS, physical component summary; MCS, mental component summary; SA, South Africa.

### Acceptability

The face validity indicated the acceptability of the SF-8 by participants based on time to completion and understanding of the questions. It took participants an average of 3 min and 30 s to complete each questionnaire (minimum of 2 min and maximum of 5 min). The entire SF-8 was accepted by the participants based on the fact that all questions were completed with limited explanation required and no complaints received from them after the completion of the SF-8. The South African Setswana version of SF-8 was composed of the same number of items and dimensions as the SF-8 South African original English version.

### Test–retest reliability

The results of the test–retest reliability for the translated Setswana and original English version of SF-8 are presented in Table 2. The reliability coefficients were moderate for the MH (*ρ* = 0.60), SF (*ρ* = 0.56) and RE (*ρ* = 0.50) domains, as well as the MCS (*ρ* = 0.50) and PCS (*ρ* = 0.45) components, but fair for the RP (*ρ* = 0.43), BP (*ρ* = 0.43), GH (*ρ* = 0.42), PF (*ρ* = 0.41) and VT (*ρ* = 0.38) domains on the translated Setswana version of SF-8. Except for the domains of GH (*ρ* = 0.51) and VT (*ρ* = 0.52) on the original English version of SF-8, all other domains and components demonstrated similar reliability values to the translated SF-8 Setswana version.

### Internal consistency

The Cronbach’s alpha coefficient was also calculated to assess the aspect of the reliability of both the Setswana and original English version of SF-8 to determine the consistency between the two versions. The Cronbach’s alpha coefficients applied for the SF-8 Setswana version were 0.87 and 0.87 for measurements taken at the first and second time points. For the original SA English SF-8 version the Cronbach’s alpha coefficients were 0.86 and 0.89 for the first and second measures, respectively.

### Concurrent validity

Spearman’s correlation coefficient ranged from a moderate (*ρ* = 0.72) to excellent (*ρ* = 0.91) relationship. These results indicate that the concurrent validity of the Setswana SF-8 was good. Total SF from the Setswana SF-8 version was significantly and highly correlated with the total SF from the original SA English SF-8 (*ρ* = 0.91, *p* < 0.001). Role emotional of the Setswana SF-8 also revealed a very high positive and significant relationship when compared with the original English SF-8 (*ρ* = 0.90, *p* < 0.001). Furthermore, encouraging significant correlations were found for RP (*ρ* = 0.72, *p* < 0.001), BP (*ρ* = 0.76, *p* < 0.001), PF (*ρ* = 0.82, *p* < 0.001), MH (*ρ* = 0.83, *p* < 0.001), VT (*ρ* = 0.82, *p* < 0.001) and the two-component summary of PCS (*ρ* = 0.83, *p* < 0.001) and MCS (*ρ* = 0.87, *p* < 0.001) when compared with the original English version of the SF-8. There were no important gender differences in the correlation coefficients of the entire item between the Setswana SF-8 and the original English version (Table 3).

**TABLE 3:** Internal consistency.

Variables	SF-8 Setswana version	SF-8 original English version
	α	α
Q1 – General health	0.87	0.86
Q2 – Physical functioning	0.84	0.85
Q3 – Role physical	0.84	0.84
Q4 – Bodily pain	0.86	0.86
Q5 – Vitality	0.85	0.83
Q6 – Social functioning	0.84	0.84
Q7 – Role emotional	0.85	0.84
Q8 – Mental health	0.86	0.85
SF-8 global score	0.87	0.86

α, Cronbach’s alpha coefficient; SF-8, Short Form-8.

## Discussion

The purpose of this study was to determine the aspect of validity and reliability of the Setswana translation of the SF-8 HRQoL survey and, alongside, to check the reliability of the SF-8 SA Standard English version in adult Setswana-speaking community dwellers living in Potchefstroom, South Africa. This study mainly described the successive steps in translating and adapting the SF-8 into Setswana psychometric properties for use among the Setswana population of SA. Furthermore, the reliability of the English version also proved to be good as a standardised measure instrument. This study showed that all the consented participants were able to read and write, which made self-administration possible.

Translation and adaptation were the first steps taken in the study, while the popular forward and backward translation method was used. The content of the questionnaire was translated into Setswana. The scale was worded in a simple and currently used language to allow for its use among Setswana people in SA. Semantic and conceptual equivalences were performed as a necessity to acquire the suitable (Guermazi et al. 2012) Setswana version of the SF-8 HRQoL, while the number of items and response modalities remained unchanged (Guermazi et al. 2012). Based on the psychometric properties, the reliability was judged satisfactory.

The alternate-form reliabilities based on the 2000 normative data were found to be 0.88 for the physical component score measure and 0.82 for the mental component score measure for the SF-8 4-week recall form. Alternate forms reported reliabilities for the health domain scales with a range from 0.70 for RE to 0.88 for BP in a large US general population sample (Quality Metric Incorporated 2011). On the other hand, 2-week test–retest reliabilities of 0.73 and 0.74 were found for the PCS and MCS measures, respectively, in a sample of recent headache sufferers using the 4-week recall survey (Quality Metric Incorporated 2011). Health domain scale test–retest coefficients ranged from 0.59 for RP to 0.70 for body pain and MH (Quality Metric Incorporated 2011).

Furthermore, the test–retest coefficient correlation between two quantitative variables was assessed using the non-parametric Spearman’s rank correlation coefficient. Based on the interpretation of the Spearman’s rank correlation coefficient, the value ranged from as low as 0.38 to as high as 0.60 for the translated Setswana version and from 0.42 to as high as 0.63 for the South African original English version. The results indicated that the relationship falls between fair and moderate. The Spearman’s rank correlation coefficients for the PCS and MCS for the translated Setswana version were 0.45 and 0.50, respectively, with fair relationships, while the South African Standard English version values were 0.63 and 0.61, respectively, with a moderate relationship (Guermazi et al. 2012).

The internal consistency of each dimension ranged from 0.84 (for PF and RP) to 0.87 (for GH) for the Setswana version, and 0.83 (for VT) to 0.86 (for GH and BP) (Table 3). The result from the internal consistency of the translated Setswana version was considered high in that the Cronbach’s alpha coefficient was well above 0.7, which was recommended for group comparison. These results were consistent with the results found in Roberts et al.’s (2008) and Guermazi et al.’s (2012) studies.

In this study, the research findings indicate higher concurrent validity for the Setswana version of SF-8. This study was in line with the study of Roberts et al. (2008), who tested the validity and reliability of the SF-8 with a conflict-affected population in northern Uganda, among 1206 adults in camps for internally displaced persons aged 18 years and above. The results revealed that the SF-8 was a reliable and valid instrument that can be used to assess HRQoL. Findings from the present study also corroborated the findings of Guermazi et al. (2012), who studied the translation in Arabic and validated the SF-36 (the SF-8 is a shorter version of the SF-36) quality of life index, in 130 participants (50 healthy subjects, 80 subjects with chronic disease [40 patients with bipolar disorders, and 40 patients with chronic renal failure]) in a Tunisian Arabic population aged between 16 and 80 years. The results showed that the translated and adapted scale had good and acceptable reliability and validity. Undeviating contrasting of findings from the present study with previous studies should be made with mindfulness, because the majority of previous studies were conducted in a different socio-economic background and settings than the present study.

## Strength and limitations

One of the main strengths of this study was that this was the first time the South African English version of the SF-8 was translated into South African Setswana for use in SA. Because this study represented the first of its kind, more studies need to be conducted to establish the validity and reliability of the Setswana SF-8 version. The main limitation of this study was the use of a small sample size. The small sample size could reduce the statistical power and limit generalisation of the findings. In addition, the non-evaluation of construct validity could be considered another limitation of the study. The acceptability of the SF-8 in this population was not sufficiently studied and is considered a limitation that should be addressed in future research. Issues related to large sample size and the other aspect of validity should be considered in future studies.

## Conclusion

In conclusion, we translated the SF-8 index into Setswana and adapted it to suit the Setswana people. This study found that the Setswana SF-8 version was feasible, acceptable and had acceptable concurrent validity (Table 4) and fair to moderate evidence of test–retest reliability for assessing the HRQoL in an adult Setswana population. Social functioning demonstrated an excellent concurrent validity but only moderate test–retest reliability. The translated Setswana version of the SF-8 for the Setswana people was found to be a valid and reliable instrument for determining HRQoL in community-dwelling persons. More studies are needed to confirm such an hypothesis with a large population sample.

**TABLE 4:** Concurrent validity of Setswana Short Form-8.

Setswana SF-8 vs. original SF-8	Total (*n* = 60)	*p*	Women (*n* = 34)	*p*	Men (*n* = 26)	*p*
	*ρ*		*ρ*		*ρ*	
Q1 – General health	0.83*	0.001	0.90*	0.001	0.73*	0.001
Q2 – Physical functioning	0.82*	0.001	0.91*	0.001	0.70*	0.001
Q3 – Role physical	0.72*	0.001	0.76*	0.001	0.70*	0.001
Q4 – Bodily pain	0.76*	0.001	0.82*	0.001	0.70*	0.001
Q5 – Vitality	0.82*	0.001	0.85*	0.001	0.83*	0.001
Q6 – Social functioning	0.91*	0.001	0.91*	0.001	0.94*	0.001
Q7 – Role emotional	0.90*	0.001	0.98*	0.001	0.80*	0.001
Q8 – Mental health	0.83*	0.001	0.90*	0.001	0.80*	0.001
PCS	0.83*	0.001	0.84*	0.001	0.81*	0.001
MCS	0.87*	0.001	0.90*	0.001	0.84*	0.001

PCS, physical component summary; MCS, mental component summary; SF-8, Short Form-8; *ρ*, Spearman’s correlation coefficient between Setswana SF-8 and original SF-8.

* , Correlation is significant at *p* ≤ 0.01 level (two-tailed).
